# Biophysical Studies of the Induced Dimerization of Human VEGF Receptor 1 Binding Domain by Divalent Metals Competing with VEGF-A

**DOI:** 10.1371/journal.pone.0167755

**Published:** 2016-12-12

**Authors:** Jean-François Gaucher, Marie Reille-Seroussi, Nathalie Gagey-Eilstein, Sylvain Broussy, Pascale Coric, Bili Seijo, Marie-Bernard Lascombe, Benoit Gautier, Wang-Quing Liu, Florent Huguenot, Nicolas Inguimbert, Serge Bouaziz, Michel Vidal, Isabelle Broutin

**Affiliations:** 1 UMR 8015 CNRS - Université Paris Descartes, Faculté de Pharmacie, Sorbonne Paris Cité, Paris, France; 2 UMR 8638 CNRS - Université Paris Descartes, Faculté de Pharmacie, Sorbonne Paris Cité, Paris, France; 3 Centre de Recherche Insulaire et Observatoire de l’Environnement USR CNRS 3278 CRIOBE, Université de Perpignan Via Domitia, Perpignan, France; 4 UF Pharmacocinétique et Pharmacochimie, hôpital Cochin, AP-HP, Paris, France; Russian Academy of Medical Sciences, RUSSIAN FEDERATION

## Abstract

Angiogenesis is tightly regulated through the binding of vascular endothelial growth factors (VEGFs) to their receptors (VEGFRs). In this context, we showed that human VEGFR1 domain 2 crystallizes in the presence of Zn^2+^, Co^2+^ or Cu^2+^ as a dimer that forms *via* metal-ion interactions and interlocked hydrophobic surfaces. SAXS, NMR and size exclusion chromatography analyses confirm the formation of this dimer in solution in the presence of Co^2+^, Cd^2+^ or Cu^2+^. Since the metal-induced dimerization masks the VEGFs binding surface, we investigated the ability of metal ions to displace the VEGF-A binding to hVEGFR1: using a competition assay, we evidenced that the metals displaced the VEGF-A binding to hVEGFR1 extracellular domain binding at micromolar level.

## Introduction

Angiogenesis is a physiological process characterized by the remodeling of the vascular tissue and the growth of new blood vessels from preexisting ones. It is an important biological process during embryonic development and tissue growth, which is limited to particular physiological phenomena in adults, such as wound healing and menstruation. Tightly controlled by pro- and antiangiogenic factors, the shift in the equilibrium under pathological conditions is associated with several human diseases, and represents a fundamental step in the angiogenic switch of malignant tumors [1].

Among the major proangiogenic cytokines, vascular endothelial growth factors (VEGFs) are essential. The five human VEGFs, VEGF-A, B, C, D, and placenta growth factor (PlGF) form homodimers, although naturally occurring heterodimers of VEGF-A and PlGF have been described [2]. VEGFs are secreted proteins that bind to transmembrane tyrosine kinase receptors (VEGFRs) primarily expressed on the surface of endothelial cells (ECs) and that induce receptor dimerization, activation through trans autophosphorylation, and assembly of the membrane-proximal signaling complex. Three receptor types have been highlighted: VEGFR3 (also called Flt-4) regulates lymphangiogenesis, VEGFR2 (KDR/Flk-1) is the primary proangiogenic receptor, whereas VEGFR1 (Flt-1) has been proposed as a regulator of VEGFR2 (for review, see [3]). Furthermore, alternative splicing or proteolytic activity of membrane VEGFR1 generates a soluble form (sFlt-1) of the extracellular domain (ECD), which sequesters VEGF-A [4]. This soluble ECD is constituted of 6 of the 7 immunoglobulin-like fold domains, missing the membrane proximal domain d7 present in the full-length receptor.

Angiogenesis is also associated with several trace elements, either exogenous or endogenous, that play critical roles in angiogenesis events. Some transition metal cations (Co^2+^, Cu^2+^, Ni^2+^, Cd^2+^) up-regulate angiogenesis and attenuate cell apoptosis in several human and murine Endothelial Cells (ECs) models (for reviews, see [5, 6]). Among these metal ions, Cu^2+^ is active at physiological concentrations [7] and its activity is correlated with VEGFR1 signaling. An excess of copper appears to be an essential co-factor for angiogenesis, and elevated levels of copper are found in plasma and malignant tissues in many types of human cancers, including prostate, breast, colon, lung, and brain [8, 9]. In contrast, Zn^2+^, that is the second most abundant metal in plasma, can dramatically reduce the expression of proangiogenic factors such as interleukin-6, and -8, VEGF, and metallopeptidase-9, while it can promote the production of antiangiogenic factors such as endostatin [5].

Anti-angiogenic targeted therapies are employed to fight cancer or age related macular degeneration. VEGFs and receptors are the main targets used in clinical practice. The pharmaceutical molecules in use are small compounds inhibiting the tyrosine kinase activity (Sunitinib (PFIZER) and Sorafenib (BAYER)), or recombinant proteins that block VEGF-VEGFR interactions, i.e. antibodies (Bevacizumab (ROCHE) and Ranibizumab (GENENTECH)) and a soluble receptor-IgG fusion protein (Aflibercept (REGENERON PHARMACEUTICALS)). Other smaller molecules preventing the ligand-receptor interaction are currently under development [10–16]. However, the susceptibility of angiogenesis to trace elements is an underestimated difficulty in the evaluation of new compound effects on angiogenesis, in both *in vitro* or *in cellulo* tests. Many organic molecules may indeed unintentionally carry metals used as catalysts for chemical reactions, and physiological or biochemical tests of new compounds may reflect the effects of these metals.

In this study, we showed the presence of a metal site at the dimer interface of X-ray structures of human VEGFR1 domain 2 (hVEGFR1d2) solved in the presence of different divalent metal ions.

The dimer interface is included in the VEGF-A recognition site, which suggests a possible dimerization mechanism via the metal, which might be competitive to VEGF-A binding.

To address this question, we first investigated the behavior of domain 2 in solution in the presence of several metals, by SAXS, NMR and analytical size exclusion chromatography. The structural reliability of our hypothesis was verified by building a dimer model of the full hVEGFR1 binding site, encompassing domains 2 and 3 (hVEGFR1d2d3), which is able to accommodate a metal ion without structural steric hindrance.

Finally, the ability of metals to displace the binding of VEGF-A for the full ECD of hVEGFR1 was tested in a biochemical assay.

## Results

### Crystallization of the hVEGFR1d2 in the presence of divalent transition metal cations

Crystallization attempts of hVEGFR1d2 were first performed in the presence of previously developed potential ligands. Although the presence of a ligand was not observed in the crystal structure, we observed the unexpected presence of one metal ion characterized as Zn^2+^ using X-ray fluorescence spectrometry. Therefore, because of the metal ions importance in the regulation of angiogenesis, we crystallized hVEGFR1d2 in the presence of several other divalent transition metal cations (Co^2+^, Cu^2+^, Cd^2+^, Mn^2+^ or Ni^2+^). Diffraction-quality crystals were obtained for three crystal forms: 1) hVEGFRt1d2 containing Zn^2+^ that crystallized in the orthorhombic *C*222_1_ space group, 2) hVEGFR1d2 that crystallized in the primitive *P*1 space group in the presence of CoCl_2_ and 3) hVEGFR1d2 that crystallized in the *I*222 space group in the presence of CuSO_4_.

### Overall crystal structure

We determined the crystal structures using molecular replacement and as search model, the hVEGFR1d2 structure issued from the complex formed between hVEGFR1d2 and the truncated VEGF-A_13-109_ (later called tVEGF-A) (PDB ID: 1FLT) [17]. The crystallographic parameters of the refined structures are summarized in Table 1. The structures of the *P*1, *C*222_1_ and *I*222 crystal forms were determined with four molecules, one molecule and three molecules in the asymmetric unit respectively. Thus, we obtained eight independent structures very similar to each other, exhibiting a rmsd comprised between 0.29 and 0.97 Å calculated on the Cα atoms depending on the superimposed pairwise. The structures exhibited an immunoglobulin-like β-sandwich fold that was typical of I-set domains, with the exception of the loop consisting of residues 137–142. This loop formed a bulge away from the core domain structure and was involved in important hydrophobic interactions with the ligands. In our structures, its backbone adopted a conformation similar to the one found in the structures of hVEGFR1d2 in complex with tVEGF-A (1QTY) [18] and with PlGF-1 (1RV6) [19], but significantly different from the alternative conformation observed in tVEGF-A/hVEGFR1d2 (1FLT) and VEGF-B/hVEGFR1d2 (2XAC) [17, 20] complexes or in the apo form (1QSZ) [18]. This supported the hypothesis of an induced fit adaptation of this loop upon ligand binding proposed by Starovasnik *et al*. [18].

**Table 1:** Crystal data collection and refinement statistics.

	*Cobalt* (4CL7)^b^	*Zinc* (4CKV)^b^	*Copper* (5ABD)^b^
Space group	*P* 1	*C* 222_1_	*I* 222
Beam line (ESRF)	ID23-EH2	ID29	ID23-EH2
Unit cell (Å, °)	27.75 41.71 94.69,	95.73 102.39 27.47,	62.55 66.19 176.48,
	87.05 82.25 73.03	90 90 90	90 90 90
Wavelength (Å)	0.8726	0.9787	0.9762
Resolution range (Å)^a^	19.47–2.00	47.87–2.05	43.97–2.00
	(2.07–2.00)	(2.13–2.05)	(2.10–2.00)
Unique reflections ^a^	26362 (2596)	8564 (737)	25367 (2503)
Data redundancy ^a^	1.8 (1.8)	3.9 (2.3)	6.3 (6.0)
Completeness (%)^a^	97.07 (96.58)	96.88 (83.85)	99.8 (99.2)
Mean I/sigma(I) ^a^	10.74 (3.01)	15.46 (3.97)	15.30 (2.06)
Wilson B-factor (Å^2^)	17.58	27.29	35.3
R-sym (%)^a^	6.9 (33.0)	5.3 (16.5)	6.9 (80.0)
Number of molecules in AU	4	1	3
Matthews coeff. (Å^3^/Da)	2.36	3.06	2.91
Solvent (%)	47.9	59.8	57.8
R-factor (%)^a^	16.75 (19.98)	16.28 (19.20)	17.85 (28.03)
R-free (%)^a^	23.32 (26.44)	20.53 (28.76)	22.16 (32.65)
Asymmetric Unit composition			
Protein residues	372	94	283
Alternate positions	0	5	4
Metal ion	5 Co	0.5 Zn	4.5 Cu, 2 Na
Sulfate	0	0	2
Ethylene glycol	5	4	0
Water	281	81	224
RMS (bonds, Å)	0.006	0.007	0.008
RMS (angles, °)	0.98	1.06	1.10
Ramachandran favored (%)	95	95	93
Ramachandran outliers (%)	0	0	1.1
Clash score	10.38	6.61	2.35
Coordinate error (Å)	0.26	0.22	0.23
Average B-factor (Å^2^)	24.40	29.70	48.40
Macromolecule	23.40	28.30	48.10
Solvent	33.40	42.30	47.70

^a^ Statistics for the highest-resolution shell are shown in parentheses

^b^ A single crystal was used for each data set

### A metal ion site at the interface of the hVEGFR1d2 dimer

**Fig 1:**
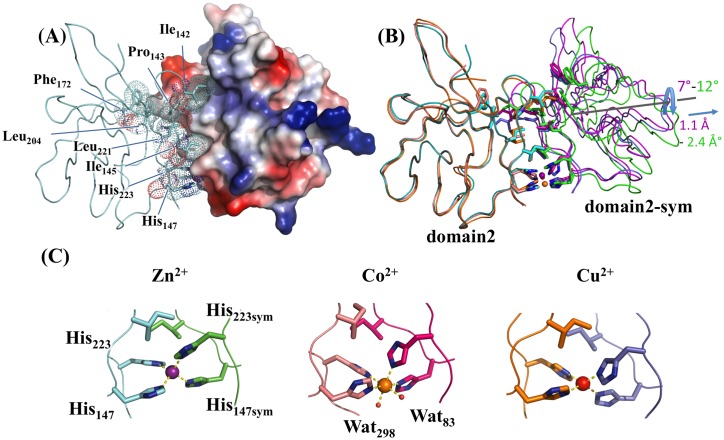
Crystal structure of hVEGFR1d2 homodimers. Zn^2+^-dimer (light blue and green) (4CKV), the Co^2+^-dimer (salmon and magenta) (4CL7) and the Cu^2+^-dimer (orange and purp(A) Interface of the Zn^2+^-induced dimer. The hVEGFR1d2 buried side chains from one monomer are represented as stick surrounded by a dot surface. The second monomer is represented by its surface conventionally colored according to the electrostatic potential. (B) The crystal le) (5ABD) were superimposed on one of the two molecules (domain2). The second molecule of the three dimers are represented on the right side: Zn^2+^-domain2-sym is rotated by 7° and translated by 1.1 Å in regard to Co^2+^-domain2-sym. The Zn^2+^-domain2-sym is rotated by 12° and translated by 2.4 Å in regard to Cu^2+^-domain2-sym. However, the residues in contact at the interface remain unchanged and their side chains are shown as sticks. (C) Metal coordination for Zn^2+^, Co^2+^ and Cu^2+^.

In the three different crystal forms the molecules were organized in similar sets of dimers related by 2-fold rotational axis passing through a metal ion: In the *C*222_1_ crystal form (4CKV), a Zn^2+^ ion lied on a 2-fold axis and coordinated two symmetrically related molecules. The Zn^2+^ ion adopted a regular tetrahedral coordination geometry and was coordinated through the Nε atoms of the imidazole groups of His_147_ and His_223_ (Fig 1C). In the *P*1 crystal form (4CL7), the asymmetric unit contained four molecules that were organized in two sets of two molecules coordinated by a Co^2+^ ion and were related by a nearly pure 2-fold rotational non-crystallographic axis. The Co^2+^ ion was coordinated through the Nε atoms of the imidazole groups of His_147_ and His_223_ on each domain and by two water molecules to form a square-based bipyramidal regular octahedron. Three additional Co^2+^ ions, which exhibited higher *B* factors, were present in the asymmetric unit and were also coordinated by a histidine residue (His_214_). In the *I*222 crystal form (5ABD), the asymmetric unit contained three molecules: two molecules were related by a 2-fold rotational non-crystallographic axis, and the third was related to its symmetric molecule by a crystallographic 2-fold axis. The Cu^2+^ had a tetrahedral coordination similarly to Zn^2+^. Three additional Cu^2+^ ions were also bound to the His_214_ and to the N-terminal main-chain of an adjacent molecule in the crystal packing. These last formed squared-planar arrangements.

The homodimerization buried 1096 ± 74 Å^2^ of accessible surface area (ASA) at the protein-protein interface. With the exception of His_147_ and His_223_, the residues involved were primarily hydrophobic including Ile_142_, Pro_143_, Ile_145,_ Phe_172_, Leu_204_, and Leu_221_ (Fig 1A and 1B). Superposition of one hVEGFR1d2 molecule of the Co^2+^-bound and Zn^2+^- or Cu^2+^-bound dimers resulted in a maximum rotation for the second molecule by 12° and a translation of 2.4 Å. The previously mentioned 137–142 loop adjusted its conformation to the surface of the adjacent molecule, and the interactions at the interface qualitatively remained similar in the two structures.

**Fig 2:**
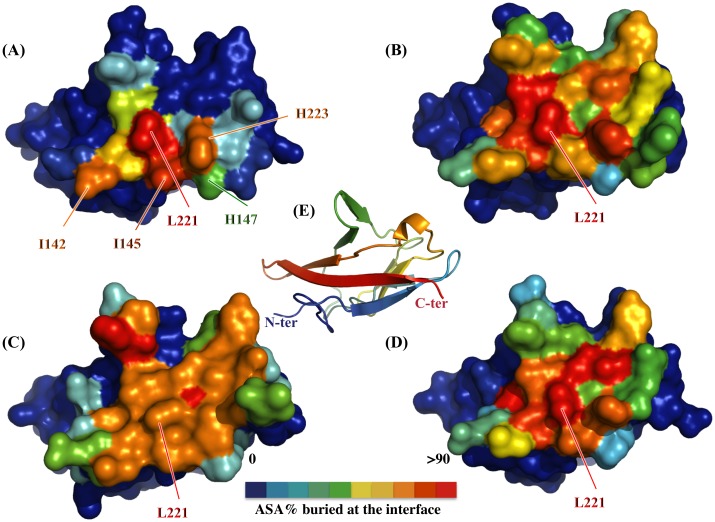
The hVEGFR1d2 dimerization interface overlapped the VEGF binding site. hVEGFR1d2 surface colored as a function of the buried surface area calculated by the program PISA, within several complexes: (A) homodimer hVEGFR1d2/hVEGFR1d2 (4CKV), (B) hVEGFR1d2/tVEGF-A (1FLT), (C) hVEGFR1d2/VEGF-B (2XAC), (D) hVEGFR1d2/PlGF (1RV6); (E) ribbon representation of the hVEGFR1d2 in the identical orientation. The homodimer interaction surface (A) mimics an important part of the hVEGFR1-ligand interaction surface (B, C, D), with a major contribution of Leu_221_.

The three crystal structures were analyzed using PISA, which is designed to identify stable complexes in crystal packing and their likelihood of representing biological units (Fig 2A). All of the complexes were assessed as stable. The mean estimation for *ΔG* dissociation was 12.7 kcal.mol^-1^, 58.1 kcal.mol^-1^ and 18.4 kcal.mol^-1^ for (hVEGFR1d2)_2_-Co^2+^, -Zn^2+,^ and -Cu^2+^ respectively. This estimation indicated that the interfaces between each pair of monomers were hydrophobic and interlocked, without unmatched electron donor or acceptor, suggesting that stable macromolecular complexes reinforced by metal chelation existed in solution. When compared to the hVEGFR1d2/VEGFs structures, all of the buried residues at the dimer interface belonged to the core of the VEGF-interacting surfaces (Fig 2B, 2C and 2D). Consequently, the dimerization that occurred in the presence of a divalent cation could be competitive with the binding of VEGFs to hVEGFR1.

### Small angle X-ray scattering (SAXS) analyses indicates metal-induced dimerization in solution

**Fig 3:**
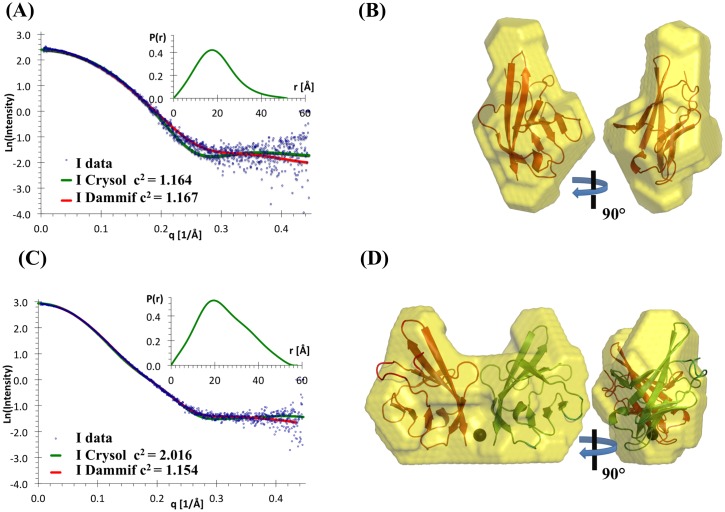
SAXS analysis of hVEGFR1d2 in the absence (A, B) and presence (C, D) of CoCl_2_. (A) and (C): scattering intensities with the CRYSOL and DAMMIF fit, and pair-distribution function (P(r)); (B) and (D): corresponding SAXS *ab initio* DAMMIF shapes. The cobalt ion is shown as a black sphere on (D).

To verify that the homodimer observed in the crystal forms existed in solution, we performed SAXS analysis of hVEGFR1d2 in the presence of 10 mM CoCl_2_ or after thorough removal of all traces of divalent cations. In both cases low-resolution SAXS data and Guinier analysis indicated the absence of aggregation, however the high-resolution data dispersion was higher in the absence than in the presence of Co^2+^ (Fig 3A and 3C). Molecular weights were estimated using Guinier analysis, pair function analysis, BSA calibration and the method of Fischer [21] (Table 2). The SAXS results unambiguously indicated that 10 mM Co^2+^ induced a doubling of the molecular weight and a significant increase of the radius of gyration, confirming the Co^2+^-induced dimerization of the hVEGFR1d2 domain in solution. Theoretical data calculated by CRYSOL fitted well with data measured in the presence of Co^2+^ (hVEGFR1d2 dimer crystal structure) or in the absence of Co^2+^ (hVEGFR1d2 monomer structure). In the presence of 10 mM Co^2+^, shapes obtained from DAMMIF were approximately symmetrical. Consequently, we imposed a *P*2 symmetry in the calculation of additional *ab initio* shapes. The 50 independent shapes calculated from DAMMIF, after filtration and superimposition by DAMAVER, exhibited a normalized spatial discrepancy (<NSD>) of 0.884 ± 0.088. This relatively low NSD indicated that the shapes converged toward similar results. Subsequently, the most representative shape was superimposed with the dimer structure of the Co^2+^ crystal form (4CL7), which is considered thereafter as the structure of reference, with a low spatial discrepancy (Fig 3D). In the absence of Co^2+^, an identical method was used to calculate 50 *ab initio* shapes in a *P*1 symmetry. The shapes converged to similar models, with <NSD> = 0.704 ± 0.091. However, some of these shapes were composed of a globular shape with a thin extension, for which the spatial orientation differs from one shape to the other (Fig 3B). All together, these results may correspond to a mixture of monomers with a minority of dimers and suggested that, even in the absence of divalent metals, a minor fraction of hVEGFR1d2 exhibited a weak propensity to dimerize in solution. In conclusion, the SAXS results were consistent with the determined homodimer crystal structures.

**Table 2:** SAXS data recording and analysis.

[CoCl_2_] (mM)	0	10
**Data-collection parameters**		
beam line (ESRF)	BM29	BM29
detector	Pilatus 1M	Pilatus 1M
Wavelength (Å)	1.008	1.008
q range (Å^-1^)	0.0039–0.4624	0.0039–0.4624
Exposure time / frame (s) (protein / buffer solution)	2.0 / 2.0	2.0 / 2.0
Number of averaged frames (protein / buffer solution)	10 / 20	10 / 20
Concentration range (mg.mL^-1^)	0.5–5	0.5–5
Temperature (K)	277	277
**Structural parameters** †		
I(0) (cm^-1^) [from Guinier] § #	11.32 ± 0.02	18.37 ± 0.02
I(0) (cm^-1^) [from P(r)] § ¥	11.31	18.53
R_g_ (Å) [from Guinier] #	15.08 ± 0.16	18.44 ± 0.14
R_g_ (Å) [from P(r)] ◊	15.10 ± 0.05	18.77 ± 0.02
D_max_(Å) ¥	51.7	57.6
Porod volume estimate (Å^3^) ◊	14635	25938
Dry volume estimated from sequence (Å^3^) ∂	13080	26050
**Molecular-mass determination**		
Partial specific volume (cm^3^.g^-1^)‡	0.749	0.749
Contrast (Δρ × 10^10^ cm^-2^) ‡	2.70138	2.6889
Molecular Mass M_r_ from I(0) (Da) ◊	12100	21500
Calculated monomeric M_r_ from sequence (Da)	11006	22012
**Software employed**		
Data reduction and processing	PRIMUS	PRIMUS
Ab initio analysis	DAMMIF	DAMMIF
Validation and averaging	DAMAVER	DAMAVER
Computation of models intensities	CRYSOL	CRYSOL
Superposition of models	SUPCOMB	SUPCOMB
Three-dimensional graphic representations	PyMOL	PyMOL

^†^ Reported for 5mg.mL^-1^ measurements § intensity on relative scale

^‡^ calculated from VolSpec software (Javier Perez and Jean-Noël Lesdema, unpublished)

^◊^ calculated from [21]

^#^ calculated from [22],

^¥^ calculated from [23],

^∂^ calculated from [24]

### Metal ion binding and reversible dimer formation probed by NMR

Copper, zinc and cobalt have a spin of 3/2, 5/2 and 7/2 respectively. In principle, these nuclei are observable by NMR, but the existence of a quadrupole moment causes a significant broadening of the signal that renders impossible their observation. However, Cd^2+^, a metal with a spin ½, is sensitive to NMR spectroscopy and zinc-to-cadmium (^113^Cd) replacement [25] may provide detailed information on the binding of the metal ion to the protein and the resulting dimerization, using ^113^Cd chemical shift perturbation mapping [26]. The ^113^Cd chemical shifts depend on the nature and on the space organization of the metal ligands [25, 27]. However, attempts to observe a signal of the ^113^Cd in interaction with the unlabeled hVEGFR1d2 were unsuccessful due to an intermediate exchange between the free form of the metal and its bound state to the protein.

**Fig 4:**
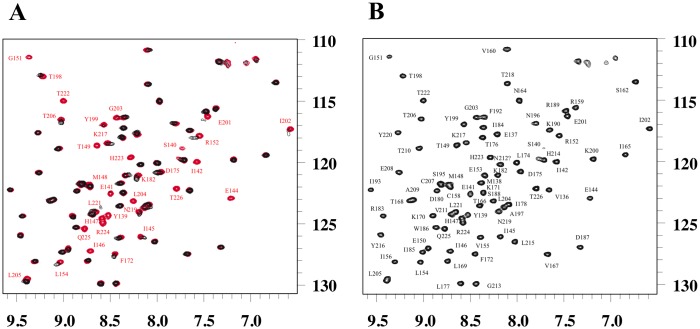
^1^H-^15^N-TROSY NMR spectra of the hVEGFR1d2 domain. (A) Overlay of the TROSY spectra of the hVEGFR1d2 at the concentration of 150 μM in the absence (red) and presence (black) of cadmium (1.8 eq). The full spectra are shown except for residues Phe_135_ and Trp_186_, with resonances at 5.6 ppm and 10.5 ppm, respectively. A general line broadening effect is observed following the addition of the divalent cation due to the dimerization of the hVEGFR1d2 and peaks of residues at the interface completely disappear due to the conformational exchange on an intermediate NMR timescale. (B) EDTA has been added (1.8 eq, 270 μM) to the precedent mixture. EDTA chelates cadmium ions, leading to the disruption of the dimer and the reemergence of the previously missing resonances characterizing the interface.

Two-dimensional ^1^H-^15^N transverse relaxation optimized spectroscopy (TROSY) [28, 29] experiments were performed using the uniformly ^15^N-labeled hVEGFR1d2 at a concentration of 150 μM for which 100% of the assignments were known. The TROSY spectra in the absence and in the presence of Cd^2+^ (50 to 270 μM) were compared and the chemical shift perturbations were analyzed (Fig 4A). In the absence of significant conformational modifications, this method allows the detection of variations in the electronic environment of the protein backbone amides that occur upon binding of the metal ion and upon dimerization.

Chemical shift perturbations were directly observed in the ^1^H-^15^N TROSY NMR spectra and several resonances of the protein exhibited a drastic line width broadening or a disappearance of the resonance associated with the addition of cadmium (Fig 4A). This well-known phenomenon is due to an intermediate exchange [30] on the NMR timescale upon cadmium binding and hVEGFR1d2 dimerization. The rate of exchange between the monomeric (M) and dimeric (D) states of the protein is equivalent to the frequency difference between the two states (k_ex_ ≈ | Ω_M_− Ω_D_ |). The broadening of the signal was so extreme that several resonances could not be detected any more in the spectrum in particular those of amino acids involved at the dimer interface. Thus it was impossible to use the chemical shift perturbation information to analyze the relative binding affinity and the stoichiometry in solution of Cd^2+^ ions. By cons, we focused on the analysis of the NMR peak volumes according to the fraction of added Cd^2+^ in the sample.

A global line broadening due to dimerization upon cadmium ion binding by histidines His_147_ and His_223_ was observed in the ^1^H-^15^N TROSY spectra and nearly 30% of the peak volumes was lost upon addition of 0.3 equivalents of Cd^2+^ (Fig 4A). This line broadening is associated with the increase of the molecular weight of the observed system and not to the intermediate exchange phenomenon that impacts the residues at the interface.

**Fig 5:**
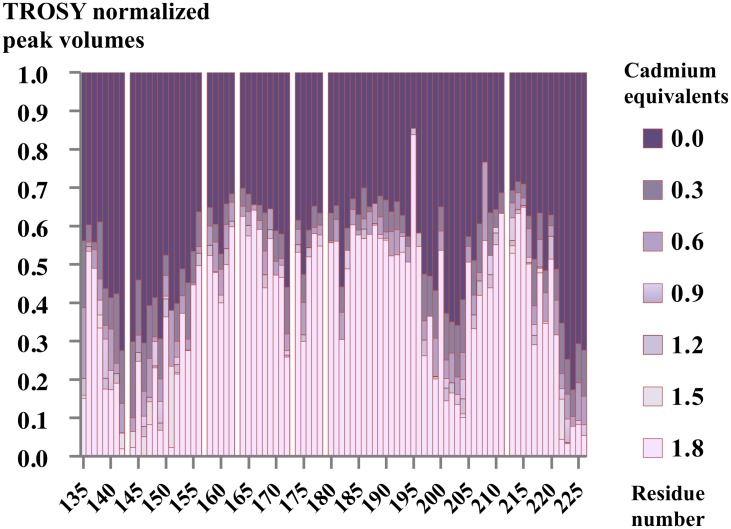
Peak volume evolution of the 1H-15N TROSY with increasing concentrations of CdCl_2_. The cadmium concentration was increased from 0 to 1.8 equivalents. The peak volumes are normalized against the highest peak volume in the TROSY experiment without cadmium. No bar indicates either the presence of proline or a residue (Asn_212_) that could not be unambiguously identified on the spectrum. Extremely perturbed peaks following cadmium addition identify three principal regions potentially involved in dimerization: Tyr_139_-Gly_151_, Ala_197_-Leu_204_ and Leu_221_-Thr_226_.

The residues involved in the dimer formation in solution were identified by following the decrease or the disappearance of the peaks at different concentrations of cadmium from 0 to 1.8 eq. (Fig 5) and three main areas were highlighted. The first one in the N-terminal region contained residues Tyr_139_-Ile_142_, Glu_144_, Ile_146_, His_147_, Thr_149_, and Gly_151_, a second region included residues Glu_201_-Leu_204_, and a third one in the C-terminal region encompassed residues Thr_222_-Thr_226_ (Fig 5). The variation of the peaks volumes was almost completed at around 0.6 eq. of cadmium that allows to roughly evaluate the stoichiometry of the interaction between the cadmium and the protein (Fig 5).

The peak volumes in the ^1^H-^15^N TROSY spectra undergoing the higher perturbations upon addition of cadmium were mapped onto the crystal structure of the dimeric hVEGFR1d2 determined in this study (S1 Fig). Among the different identified regions, Tyr_139_-Gly_151_ and Thr_222_-Thr_226_ contain His_147_ and His_223_ respectively, involved in both metal ion recognition and dimerization.

To determine whether dimerization is reversible, EDTA (270 μM), a metal ion chelator, was added to the NMR sample containing 150 μM hVEGFR1d2 and 270 μM CdCl_2_. The obtained spectra (Fig 4B) indicated that the divalent metal cation was neutralized upon the addition of EDTA, and the spectra recovered the original resonances that were present in the absence of cadmium. Consequently, the dimer interface identified in solution was very close to that identified in the crystal structure, and dimerization was clearly due to coordination of the metal ion by these two histidines from both monomers.

### hVEGFR1d2d3 molecular modeling

After demonstrating the metal induced dimerization of domain d2 in solution, the question occurring was the capacity of the full VEGF binding site, i.e. the d2d3 domains of hVEGFR1, to dimerize in solution in the presence of a metal. Consequently, we modeled the hVEGFR1d2d3 and simulated its behavior in solution by molecular dynamics (MD).

First, an atomic model of domain d3 was built by homology modeling from the VEGFR2d3 structure (3S37). The degree of residues conservation between hVEGFR1d3 and VEGFR2d3 (36%) resulted in a set of very similar models, at the exception of the 270–282 segment that displayed several alternative orientations because of a lower degree of homology. The resulting model of domain d3 was connected to our reference crystal structure of hVEGFR1d2 issued from the P1 crystal structure (4CL7), oriented as in the VEGFR2d2d3/VEGF-C structure (2X1X).

**Fig 6:**
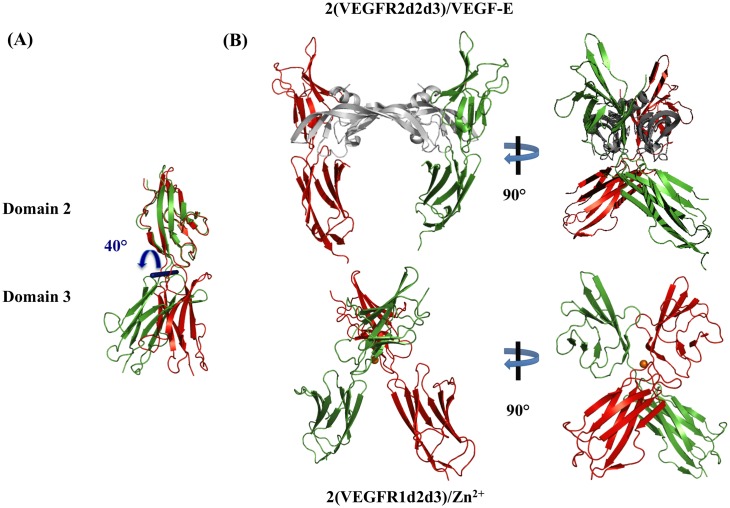
hVEGFR1-d2d3 structural modeling. (A) hVEGFR1d2d3 model after MD simulation: two extreme conformers observed during the MD simulation are superimposed on domain 2 to illustrate the 40° hinge motion. (B) Top: crystal structure of the VEGFR2-d2d3/VEGF-E complex (3V6B). Bottom: Zn^2+^-dimerized hVEGFR1d2d3 model after MD simulation. The Zn^2+^ ion is shown as an orange sphere. Equivalent orientations of domain 3 are shown in identical colors (red or green).

MD simulation was performed on the resulting monomer using the Gromacs 4.5.5 package, in explicit water and NaCl, for a time period of 1 ns at 300 K. During the MD, the total energy fluctuated weakly (-1223570 ± 700 kJ.mol^-1^), which indicated a good stability of the system. Calculation of the RMS displacements on all atoms showed that the main fluctuations were equally distributed in the loops of both domains, and that the core of each domain was stable. The resulting model conserved a good stereochemical quality (Structural Analysis and VErification Server—SAVES). The only negative was the low backbone Z-score (Z = -6.4), despite a good geometry. The regions presenting a slightly poorer geometrical quality were the 174–176 and 208–212 segment of d2, and the C-terminus (311–321) of d3 oriented toward the fourth domain in the full receptor context. During the MD, a large hinge movement around residues 223–226 was observed with a relative orientation between the two domains fluctuating up to 40° without significant energy variation (Fig 6A) (DynDom) [31]. To simulate the Zn^2+^-dimerized hVEGFR1d2d3, two molecules of the built hVEGFR1d2d3 model were then superimposed on the crystallographic dimer of hVEGFR1d2. Two MD simulations were performed on this resulting dimer model, with and without the presence of Zn^2+^. The MD procedures were identical to the previous one, but continued during a total of 2 ns. All along MD simulations, the d2 domains remained bound via the hydrophobic interface previously described in the crystal structures. However, the Zn^2+^ stabilized the crystallographic d2 interface, while in the absence of metal, the two d2 domains slightly rotated relative to each other. Moreover, we noticed that the Gln_225_ side chains of both molecules moved in the vicinity of Zn^2+^, adding two bonds to the metal, and forming a square based bi-pyramid octahedral coordination. Despite this supplementary constraint on Gln_225_, residues 224–226 still formed a hinge between the two domains, as in the MD simulation performed on the monomeric d2d3 model.

The first striking point is that the d2d3 model in the same relative orientation than that observed in the VEGFR2d2d3/VEGFs crystal structures can be dimerized in the presence of metal without steric hindrance. The second point is that, after MD simulation, the relative orientation of the two d3 in the dimer model differed from what was observed in VEGFR2d2d3/VEGF-A, -E, and -C crystal structures (3V2A, 3V6B, 2X1W respectively) [32, 33] (Fig 6B), as they faced by the opposite surfaces. Nevertheless, the d3 main axis was roughly perpendicular to the membrane surface, and similar to the direction observed in VEGFR2d2d3/VEGF complexes (Fig 6B). It suggested, on a structural point of view, that a metal-induced dimer of the full-length receptor could be anchored in the membrane, but that the relative positions of domains 3–7 were completely different than in the dimer induced by VEGF binding, probably precluding activation.

### Displacement of VEGF-A binding to the hVEGFR1-ECD by divalent cations in a competition assay

The effect of several divalent cations to disrupt the VEGF-A/hVEGFR1 interaction were evaluated using a previously described chemiluminescent competition assay that utilized biotinylated VEGF-A and recombinant human hVEGFR1-ECD/Fc dimerized by disulfide [34]. Initial screening at 30 μM revealed the ability of some divalent cations, including Cu^2+^, Pd^2+^, Ni^2+^ and Zn^2+^, to destabilize the VEGF-A/hVEGFR1 interaction. As indicated in Table 3, copper (CuSO_4_) and palladium (Pd(OAc)_2_) demonstrated the lowest IC_50_ values (0.97 and 0.36 μM, respectively). Zn^2+^ and Ni^2+^ exhibited IC_50_ values within the micromolar range. Other divalent cations exhibited little (Co^2+^ and Cd^2+^) or no effect on the competition assay (Fe^2+^ and Mn^2+^). These various effects suggested that the binding site was flexible and able to adapt to different cation size and coordination, which was consistent with the structural results. For cations of similar size, the observed differences may be due to different preferred coordination geometry [35] and ligands. The effect of counter ions was also evaluated by examining CuSO_4_, CuCl_2_, Cu(OAc)_2_, Pd(OAc)_2_, and PdCl_2_, and resulted in non-significant differences between anion species. To confirm the specific interaction of Cu^2+^ with hVEGFR1, the competition assay was performed in the presence of a metal chelator (EDTA, 1 eq.). Under these conditions, Cu^2+^ was unable to disrupt the VEGF-A/hVEGFR1-ECD interaction. These results, together with the determined crystal structures, suggested that metal ions, particularly Cu^2+^ and Pd^2+^, competed with biotinylated VEGF-A in our assay.

**Table 3:** displacement of VEGF-A binding to the VEGFR1-ECD by divalent cations in competition assay. IC_50_s and % inhibition of assayed metal complexes.

Metals	% displacement at 30 μM ^a^	IC_50_ [95% CI](μM) ^b^
CuSO_4_	83 ± 8	0.97 [0.63–1.49]
Cu(OAc)_2_	69 ± 2	1.32 [0.90–1.94]
CuCl_2_	87 ± 2	1.26 [1.02–1.55]
Pd(OAc)_2_	97 ± 3	0.36 [0.27–0.48]
PdCl_2_	93 ± 1	0.25 [0.19–0.31]
NiSO_4_	75 ± 1	3.54 [2.67–4.70]
ZnSO_4_	61 ± 2	5.94 [4.80–7.37]
CoSO_4_	44 ± 1	N/A^c^
CoCl_2_	40 ± 2	47.11 [35.33–62.81]
CdCl_2_	38 ± 4	47.97 [32.55–70.69]
FeSO_4_	13 ± 5	N/A
MnSO_4_	0	N/A
Na_2_SO_4_	0	N/A

^a^ Activity corresponds to the percentage of biotinylated VEGF-A_165_ displaced by 30 μM of metal on the whole extracellular domain (ECD: d1-d7) of VEGFR1.

^b^ Inhibitory concentration able to displace 50% of the biotinylated VEGF-A_165_ binding on VEGFR1 (ECD).

^c^ Problem of insolubility

### hVEGFR1d2 dimerization using analytical size exclusion chromatography

**Fig 7:**
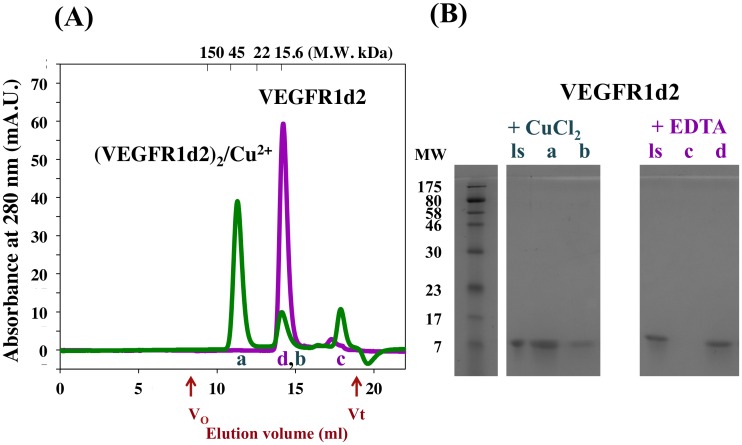
Size-exclusion chromatography reveals the Cu^2+^ induced hVEGFR1d2 dimerization. (A) hVEGFR1d2 eluted either in the presence of either 1 mM EDTA (Purple curve) or 1 mM CuSO_4_ (Green curve). The chromatogram revealed a single peak in the presence of 1 mM EDTA (d), and two major peaks corresponding to the monomer (b) and the dimer (a) in the presence of Cu^2+^. A third peak (c) consisting in Cu^2+^ ions eluted near the total volume. (B) SDS page of loaded samples and eluted peaks. hVEGFR1-d2 + CuCl_2_ (Green): Loaded sample (ls), Ve = 11.3 ml (a), Ve = 14.1 ml (b); hVEGFR1-d2 + EDTA (Purple): Loaded sample (ls), Ve = 17.3 ml (c), Ve = 14.0 ml (d).

Because the competition assay results indicated a higher effect of Cu^2+^ ions compared to Zn^2+^ or Co^2^, the Cu^2+^-induced dimerization of hVEGFR1d2 in solution was verified using analytical gel-filtration chromatography. In the presence of 1 mM EDTA a single hVEGFR1d2 peak was observed, which represents the monomer (apparent MW = 14.9 kDa). In the presence of 1 mM CuSO_4_, two peaks were eluted corresponding respectively to the hVEGFR1d2 dimer (78% of absorption, apparent MW = 27.9 kDa) and monomer (apparent MW = 15.8 kDa). SDS-PAGE analysis confirmed that both peaks consist of hVEGFR1d2 (Fig 7).

### Sequence alignment of VEGFR1d2

The residues buried at the dimer interface (Ile_142_, Pro_143_, Ile_145,_ Phe_172_, Leu_204_, and Leu_221_) are conserved or conservatively substituted in VEGFR1 (S2 Fig) through mammals. The His_147_ and His_223_ are conserved in the primate order. Nevertheless, in the Mammalia class His_147_ is often substituted by Tyr or Asn, and in the specific Pig, Bovine and Sheep species His_223_ is substituted by Ile or Leu. Consequently, the metal site that we identified in VEGFR1 is not ubiquitous, though Tyr and Asn may frequently act as metal-binder residues.

In VEGFR2 conserved Tyr and Ile residues substitute His_147_ and His_223_ respectively. In VEGFR3 His_147_ is deleted and His_223_ is substituted by Ile or Val residues. The metal binding site seems consequently not present in VEGFR-2 and -3.

## Discussion

We have determined the hVEGFR1d2 crystal structure in the presence of Co^2+^, Zn^2+^ or Cu^2+^. Contrary to the Co^2+^ and to the Cu^2+^ ions, the Zn^2+^ was not intentionally added in crystallization conditions, but was *a posteriori* identified by X-ray fluorescence. A zinc contamination arising from laboratory items cannot be excluded [36] or from the putative ligand Laxaphycin B. This cyclic peptide does not possess the peculiarity of some Zn^2+^ chelating peptides as Cys residues [37], or thiazole and oxazoline rings [38]. However the pseudofactin II, a cyclic lipo-octapeptide, has recently been shown to chelate Zn^2+^ via carbonyl oxygens from the main backbone in a 1:1 stoichiometry [39]. It might also be the case for Laxaphycin B.

The three structures are similar and reveal a homodimer with interlocked hydrophobic surfaces buried at the interface and a divalent cation chelated by four histidine residues (two from each monomer). The metal ion coordination site is octahedral for Co^2+^ and tetrahedral for Zn^2+^ and Cu^2+^. These are the most frequent coordination numbers observed in the Protein Data Bank (PDB) for each of the ions [35]. However, the analysis of non-redundant structures shows that the coordination of Cu^2+^ by three or four His residues is highly frequent and that such coordination is scarce for Co^2+^ and Zn^2+^[40]. The coordination distances for Nε His-Co, Nε His-Zn and Nε His-Cu belong to [2.20 Å; 2.49 Å], [2.19 Å; 2.34 Å], and [2.06 Å; 2.32 Å] respectively and are slightly higher than expected from a statistical analysis of the PDB (Nε His-Co^2+^ = 2.07 Å, SD = 0.12 Å (n = 752); Nε His-Zn^2+^ = 2.07 Å, SD = 0.12 Å (n = 279); Nε His-Cu^2+^ = 2.03 Å, SD = 0.10 Å (n = 47)) [40]. However, as they were not defined as covalently linked in the X-Ray refinement software, the slightly higher distances may result from the repulsive part of the Van der Waals forces.

All dimers are analyzed as stable in solution by PISA. SAXS analysis, NMR (intermediate exchange) and analytical size-exclusion chromatography verify respectively the Co^2+^-, Cd^2+^-, and Cu^2+^-induced dimerization of hVEGFR1d2 in solution. The SAXS shapes are consistent with the determined crystal structures for both monomer and homodimer. NMR analysis shows that hVEGFR1d2 dimerization is reversible using EDTA as a chelating agent. Importantly, the dimer interface involves residues that lie in the core of the hVEGFR1d2 surface that interacts with VEGF-A, VEGF-B or PlGF.

Thus, we hypothesize that some metal ions may induce the homodimerization of the full-length hVEGFR1-ECD. To evaluate this hypothesis, a model of hVEGFR1d2d3 was built, oriented as in the VEGFR2d2d3 template and positioned as observed in the Zn^2+^ hVEGFR1d2 dimer structure, followed by a GROMOS molecular dynamics simulation performed in the presence of explicit water molecules and NaCl [41]. The homodimeric model retains the d2 dimer interface and the bound Zn^2+^ ion after a 2 ns molecular dynamics simulation. We observe a movement of d3 relative to d2 that suggests that a metal-induced dimer of the full-length receptor can be anchored in the membrane, but with a modified relative position of domains 3–7, that probably precludes the tyrosine kinase domains activation (Fig 6B). The model is also compatible with the metal-induced dimerization of the hVEGFR1-ECD/Fc used in our biochemical assay.

Because the dimerization seems to interfere with VEGF binding, we investigated the ability of metal ions to displace the VEGF-A. *In vitro* competition for the whole hVEGFR1-ECD predimerized *via* Fab indicates that several divalent cations displace VEGF-A from its target with micromolar IC_50_ values. Cu^2+^ and Pd^2+^ are the most efficient divalent cations, but Ni^2+^, Zn^2+^, and Co^2+^ also exhibit an effect to a lower extent.

The current study doesn’t explore the biological relevance of the metal-induced dimerization site. However, it constitutes an interesting point, considering the numerous studies that focused on the role of metals in angiogenesis and the lack of mechanistic evidence at the molecular level. Two metals ions, copper and zinc, have been evidenced to regulate angiogenesis in physiological conditions [6]. They are the second and the third most abundant trace metals in plasma (16.5 ± 8.6 μM and 16.6 ± 6.2 μM, respectively) [42]. Plasmatic zinc is bound to and transported by albumin and transferrin, and the stock is rapidly exchangeable [43], while the major part of plasmatic copper is tightly bound to ceruloplasmin, with a low exchangeable fraction (0.5 μM) [44]. Consequently, the IC_50_ that we observed in our displacement assays are on the same order of magnitude than exchangeable Cu^2+^ and Zn^2+^ concentrations in plasma.

The molecular basis for the sensitivity to copper remains elusive and controversial: a known target is the hypoxia-inducible factor-1 (HIF-1α), which binds HIF-1β under hypoxic conditions, and promotes *vegfs* and other genes transcription [45]. However Li *et al*. demonstrate that a low Cu^2+^ level (5 μM) increases cell proliferation without increasing the VEGF expression in HUVEC and aortic cells [46, 47]. Several studies also establish that Cu^2+^ activity is mediated through the switch from VEGFR2-dependant to VEGFR1-dependant pathway (for review, see [5, 48]).

The role of zinc in a wide range of cellular processes has been well established. It plays an important role in transcription factors function, DNA repair and as antioxidant. More specifically zinc has an anti-angiogenic activity through the Zn-bound endostatin. It may also reverse gene expression of most genes modulated by hypoxia. Especially zinc supplementation was shown to be effective on HIF-1 activity, resulting in lower expression of VEGF [5]. Recently a Zn^2+^ site was also identified on the extracellular part of Neuropiline-2, of which the physiological ligand is VEGF-A, and was proposed as a regulatory site [49].

The binding equilibrium of VEGF-A toward hVEGFR1 and hVEGFR2 is an important point of the angiogenesis regulation, as the homo and heterodimerization of hVEGFR1 / R2 has been proposed to switch the activation of antagonistic transduction signaling [50–55]. Moreover, the soluble form (sFlt-1) of the VEGFR1 sequesters VEGF-A and is a well-known physiological antiangiogenic factor. In this context, the biological significance of a metal site on hVEGFR1 might be further questioned.

Finally, in addition to further investigations in metal ion functions in angiogenesis, the results presented here are useful to screen new VEGFR ligands, and may prevent false positives in biochemical assays using the hVEGFR1-ECD or hVEGFR1d2 [17, 34, 56]. Indeed, numerous compound libraries are synthetized using copper, palladium, and other transition metals as catalysts. Transition metals must be rigorously discarded in the purification steps. The metal site can also be exploited to design new therapeutic compounds that target specifically the hVEGFR1.

## Materials and Methods

### Protein expression, purification and refolding

The human hVEGFR1d2 protein (residues 132–226) was expressed and purified as previously described [13]. For SAXS analysis, all divalent metal ions were thoroughly removed with 0.25 mM EDTA, followed by three dialysis against 250 volumes of 20 mM HEPES-NaOH, pH 7.0, containing 160 mg/l of divalent cation-chelating resin Chelex^®^100 (Sigma-Aldrich, St. Louis, USA).

For ^15^N labeling, hVEGFR1d2 was expressed in *E*. *Coli* Rosetta-gami(DE3)pLysS bacterial strain (Novagen, Darmastadt, Germany), cultured in ^15^N-isotope-enriched medium (CortecNet, Voisin-le-Bretonneux, France) and purified as previously described.

### Biochemical assays

The ability of the refolded human hVEGFR1d2 domain to bind to VEGF-A_165_ was evaluated by an ELISA-type chemiluminescent assay. It was based on competition for binding to biotinylated VEGF-A_165_ of either the refolded hVEGFR1d2 or the immobilized human-hVEGFR1-ECD/Fc chimera [34].

Based on the same assay, the ability of metal ions to prevent the VEGF-A/hVEGFR1-ECD interaction was evaluated. For this study, the divalent metal salts described in Table 3 were used, and Na_2_SO_4_ was used as the control. Briefly, metal ions were preincubated for 1 hour in a 96-well microplate (96-well, high-binding white plates, Corning, New-York, USA) coated with a human VEGFR1-ECD, predimerized *via* Fab bound by a 6 amino acids linker to the His_687_ in the d7 domains (20 ng/well, R&D Systems, Abingdon, UK). Subsequently, a fixed amount of biotinylated human VEGF-A (131 pM, R&D Systems, Abingdon, UK) was incubated with the evaluated metal ions for 2 hours. After PBS/0.1% Tween 20 washing, the remaining biotinylated VEGF-A was detected by chemiluminescence using HRP-conjugated streptavidin (GE Healthcare, Little Chalfont, UK). The divalent metal ions were evaluated from 30 to 0.03 μM in the presence of 1% DMSO.

### Crystallization, structure determination and analysis

hVEGFR1d2 was crystallized using the vapor diffusion technique at 18°C and equal volumes of reservoir and protein solution. First crystals containing Zn were serendipitously obtained in the presence of a putative VEGFR ligand, the Laxaphycin B [13, 57] that revealed to be absent from the electron density. Other divalent metal cations were intentionally introduced. Diffraction-quality crystals were optimized for three crystal forms: the first crystallized in the space group *C*222_1_, from a drop containing 1 mM hVEGFR1d2, 0.1% (w/v) n-Dodecyl β-D-maltoside and 4 mM Laxaphycin B [57] in 5 mM HEPES-NaOH, pH 7.5, and a reservoir solution containing 18% (w/v) PEG 8000 and 100 mM HEPES-NaOH, pH 7.5. The second crystal form crystallized in the *P*1 space group with 1 mM hVEGFR1d2 in 5 mM HEPES-NaOH, pH 7.0, 10 mM CoCl_2_, and a reservoir containing 100 mM Bis-Tris-HCl, pH 6.5, and 15% (w/v) PEG 3350. The third crystal form crystallized in *I*222 space group from a drop containing 1.5 mM hVEGFR1d2, 15 mM Tris-HCl pH 8.1, 25 mM NaCl, 5 mM CuSO_4_, and a reservoir solution containing 19.5% (w/v) PEG 8000, 100 mM NH_4_SO_4_, and 90 mM HEPES-NaOH, pH 7.5.

Diffraction data were collected at the European Synchrotron Radiation Facility (ESRF, Grenoble, France) at the ID23-EH2 microfocus [58] and ID29 beamlines [59] and processed with the XDS X-Ray detector Software package [60]. The structure for the *P*1 crystal form was solved by molecular replacement using Phaser [61, 62] and the hVEGFR1d2 structure (1FLT) [17] as the search model and was further optimized using PHENIX [63] for refinement and Coot [64] for interactive model building. After initial rounds of refinement, two major peaks remained for each molecule in the 2F_o_-F_c_ and F_o_-F_c_ difference maps. The first peak corresponded to the cobalt ion, and the second peak corresponded to a distortion of the main chain between residues 135 and 142. An iterative omit map was calculated [63] to rebuilt this segment in the four molecules of the asymmetric unit.

The second crystal structure was solved in the *C*222_1_ space group, with one molecule per asymmetric unit. An identical procedure was performed using the previously refined model as the search model. The F_o_-F_c_ map revealed a 15 σ peak localized at a special crystallographic position on the 2-fold axis that reconstitutes the dimer. Because the nature of this atom was unknown, X-ray fluorescence spectra were performed on the PROXIMA 1 beamline of the SOLEIL synchrotron (Saint-Aubin, France) [65] (Si drift diode energy dispersive detector, RONTEC Inc.). Four spectra recorded on crystals from the same crystallization box revealed a unique anomalous signal corresponding to zinc K edge (f’ = -6.80 e^-^ and f” = 5.83 e^-^ at 9.673 keV, f’ = -6.77 e^-^ and f” = 2.33 e^-^ at 9.666 keV. Signal amplitude of the multichannel analyzer: 80 counts at 9.640 keV, 1320 counts at 9.673 keV, and 880 counts at 9.680 keV) (S3(A) Fig). The electronic density was consequently interpreted as a Zn atom, although no Zn was intentionally introduced in the crystallization drop. To evaluate the signal accuracy, a X-ray fluorescence spectrum was also recorded on the P1 crystal form, which resulted in an unique anomalous signal corresponding to cobalt K edge (f’ = -6.67 e^-^ and f” = 6.70 e^-^ at 7.727 keV, f’ = -10.32 e^-^ and f” = 3.44 e^-^ at 7.722 keV). Signal amplitude of the multichannel analyzer: 50 counts at 7.700 keV, 1450 counts at 7.727 keV, and 900 counts at 7.740 keV) (S3(B) Fig).

The third crystal structure was solved in the *I*222 space group, with three molecules per asymmetric unit. An identical procedure was performed using the previously refined model as the search model. The final refinement statistics for the three refined structures are summarized in Table 1.

Structural analysis of each dimer surface and interface was performed using the PISA server (European Bioinformatics Institute) [66].

Molecular graphics were rendered using PyMOL (Molecular Graphics System, Schrödinger, LLC).

### Small-angle X-ray scattering

We measured SAXS data of hVEGFR1d2 solutions in the absence or presence of CoCl_2_, at the BM29 BioSAXS beamline at the European Synchrotron Radiation Facility (ESRF, Grenoble, France). Frames were integrated, checked for radiation damage, and averaged using the PRIMUS 3.1 software [22]. After background subtraction, data were analyzed using the ATSAS Version 2.4.3 program package [67] and SAXS MoW software [21]. The absence of a concentration dependence of the scattering data was verified using three concentrations ranging from 1.5 to 5.0 mg/mL in the presence or absence of 10 mM CoCl_2_. The distance pair-distribution functions were calculated from the scattering curve using GNOM [23], and *ab initio* shapes were determined using DAMMIF [68]. Fifty independent shapes were aligned and filtered using DAMAVER [69]. The most representative shapes, as determined by DAMSEL, were aligned with crystal models using SUPCOMB to produce the structural representations without Co^2+^ (<NSD_best shape/other shapes_> = 0.651; NSD_best shape/hVEGFR1d2 monomer_ = 0.925) and with Co^2+^ (<NSD_best shape/other shapes_> = 0.819; NSD_best shape/hVEGFR1d2 dimer_ = 0.989). X-ray solution scattering of the monomeric and dimeric crystal structures were evaluated using CRYSOL [24] and were compared with the experimentally determined values. Mean SAXS data-collection and scattering-derived parameters are summarized in Table 2 and Fig 3.

### NMR data acquisition

All spectra were acquired at 300 K using a Bruker AVANCE 600 MHz spectrometer equipped with a 5-mm triple resonance probe and triple axis pulsed field gradients. One-dimensional ^113^Cd NMR spectra were acquired at different metal ion concentrations (from 50 to 300 μM) in the presence of the unlabeled hVEGFR1d2 at a concentration of 150 μM to observe its signal upon interaction with the d2 domain. Typical NMR samples used for the ^1^H-^15^N TROSY dimerization assays *via* metal ion binding consisted of 150 μM of uniformly ^15^N-labeled hVEGFR1d2 in 300 μL of a 20 mM phosphate buffer at pH 5.7 containing 50 μM EDTA and 10% (v/v) ^2^H_2_O. The metal was solubilized in 100 μL of H_2_O to yield a concentration of 15 mM, and 1 μL aliquots of this solution were added to the NMR sample for titration experiments until a 2.1 equivalent was reached. Chemical shift referencing of the ^1^H and ^15^N assignments was performed using previously published assignments [18]. Spectral processing and analysis were performed using TopSpin 1.3 and CcpNmr 2.1.5 [70]. Backbone spectral ^1^H-^15^N assignment was performed as previously reported, and volumes were normalized against the highest peak volume in the TROSY experiment.

### Size-exclusion chromatography

The Cu^2+^-induced dimerization of hVEGFR1d2 was confirmed using a precalibrated analytical gel-filtration column (Superdex^™^75 HR 10/300 Amersham GE Healthcare, Little Chalfont, UK). Prior to loading, samples were prepared by incubating 160 μM hVEGFR1d2 in a buffer containing 25 mM Tris-HCl, pH 8.1, 250 mM NaCl, and either 1 mM EDTA or 1mM CuSO_4_ for 30 min at 18°C. Subsequently, 50 μL of this mixture was loaded at 0.5 mL/min on the column that was equilibrated with the identical respective buffers. Elution peaks were integrated, and the corresponding fractions were analyzed on SDS-PAGE.

### hVEGFR1d2d3 modeling

hVEGFR1 domain 3 modeling was performed using I-TASSER [71]. To crosscheck its validity, other models were performed by using the structure of VEGFR2d3 as template (3S37), on two other servers, CPHmodels3.2 (http://www.cbs.dtu.dk/services/CPHmodels/) [72], and Geno3D (http://geno3d-pbil.ibcp.fr/) [73]. A fourth model was obtained from pGenThreader (http://bioinf.cs.ucl.ac.uk/psipred/) [74] using the structure of VEGFR2d2d3 (2X1W). Accuracy of models was evaluated using the Structural Analysis and VErification Server (http://nihserver.mbi.ucla.edu/SAVES/).

After validation, the best model of domain 3 was connected to the domain 2 issued from the *P*1 crystal structure (4CL7), oriented as in the VEGFR2d2d3/VEGF-C structure (2X1X).

MD simulations were then performed by using the Gromacs 4.5.5 package [41], and united atom GROMOS96 53a6 force field at 300 K, in explicit water and NaCl: the model of hVEGFR1d2d3 was energy minimized using the steepest descent, until the maximum force on the atoms was smaller than 1 kJ.mol^-1^ and convergence was obtained. Then the modeling box was fulfilled with 31700 water molecules and 250 mM Na^+^ and Cl^-^ ions, and the solvent was equilibrated by energy minimization, followed by restrained MD (5 ps). Two steps of MD were performed with initial velocity drawn from Maxwell distribution at 300K, followed by simulation of pressure by the Berendsen procedure. Then final productive MD of unrestrained system was performed for 1 ns at 300K, with an integration time step of 1 fs, and Parrinello-Rahan Pressure coupling.

After validation of the MD results with SAVES, we re-used the model to perform MD on dimerized hVEGFR1d2d3 in the presence or in the absence of a Zn^2+^ ion in the metal site. The initial orientations and interactions of dimerized d2d3 were those determined in the here solved crystal structure of hVEGFR1d2 in the presence of Zn. We tested several initial orientations of d3 in regard to d2. The MD procedure was identical to the previous one, but the MD was continued during a total of 2 ns.

## Accession Numbers

The PDB ID for the structures of hVEGFR1d2 in complex with cobalt, zinc and copper are 4CL7, 4CKV, and 5ABD respectively.

## Supporting Information

S1 Fig Mapping of the perturbed resonances volumes onto the homodimeric crystal structure of hVEGFR1d2. Amino acids with drastic volume decrease after addition of cadmium have been colored in red on the structure. The cadmium ion is represented as a space filling model and colored in purple. The cadmium bridges the two monomers of hVEGFR1d2 by binding histidines His_147_ and His_223_ of each monomer. Three regions can be identified, Ile_142_-Thr_149_, Glu_201_-Leu_204_ and Thr_222_-Arg_224_, involved either in metal recognition and or in dimer formation.
(TIF)

S2 Fig Sequence alignment for the VEGFR1d2 (flt1) in the class Mammalia. The residues encompassing the dimerization site are in green boxes and the His_147_ and His_223_ homologous are in red boxes.
(TIF)

S3 Fig X-ray fluorescence spectra of hVEGFR1d2 crystals. (A) hVEGFR1d2 crystallized in *C*222_1_ space group in the presence of 4 mM Laxaphycin B. (B) hVEGFR1d2 crystallized in *P*1 space group in the presence of 10 mM CoCl_2_. The spectra revealed a unique anomalous signal for each crystal form that corresponds to zinc K-edge (A) or to cobalt K-edge (B).
(TIF)
