# Supplementary figures and images for: Biophysical Studies of the Induced Dimerization of Human VEGF Receptor 1 Binding Domain by Divalent Metals Competing with VEGF-A
Source: PLoS One. 2016 Dec 12;11(12):e0167755. doi: 10.1371/journal.pone.0167755 (PMC5152890; doi:10.1371/journal.pone.0167755)

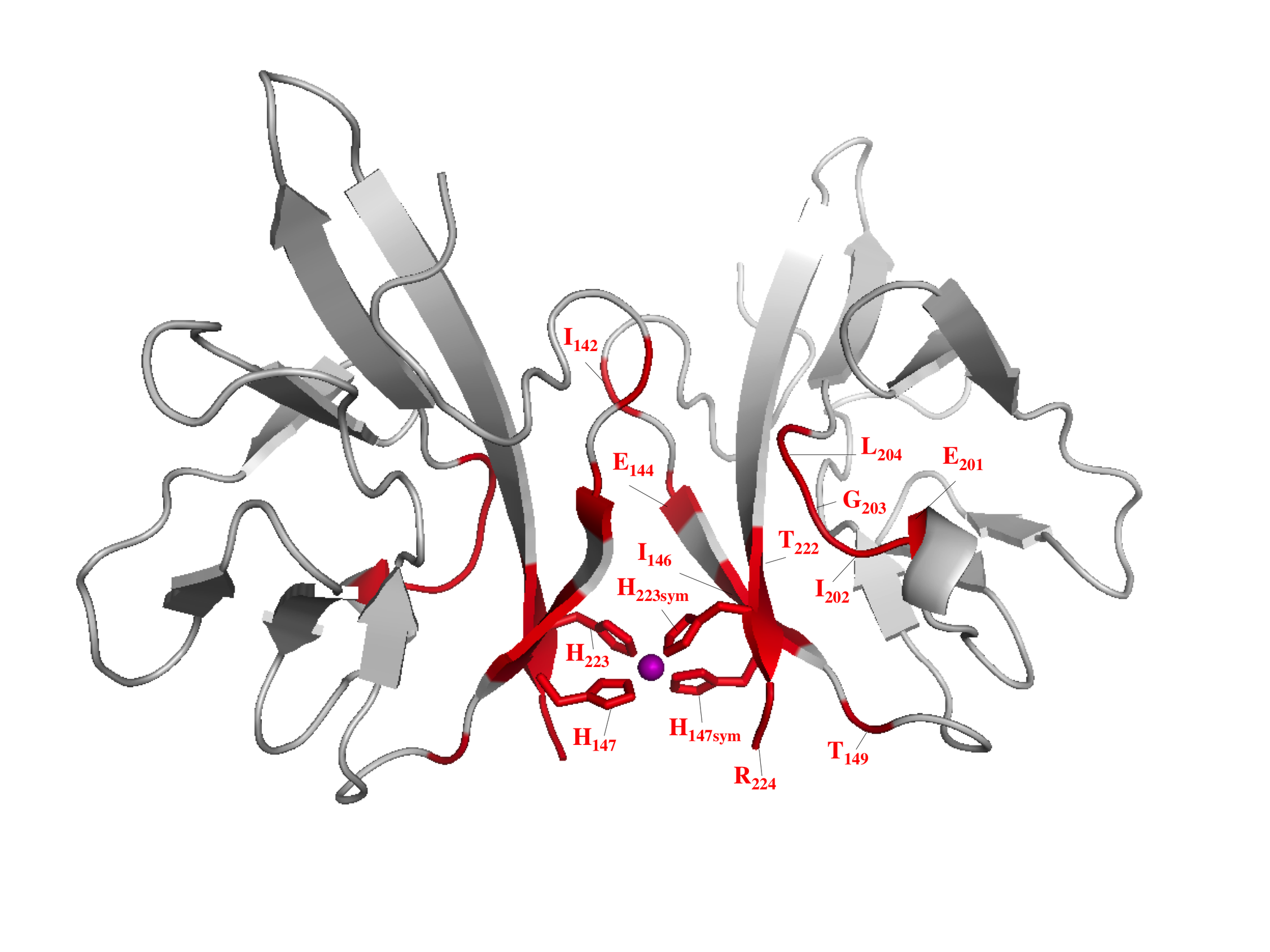

Supplement: S1 Fig — Amino acids with drastic volume decrease after addition of cadmium have been colored in red on the structure. The cadmium ion is represented as a space filling model and colored in purple. The cadmium bridges the two monomers of hVEGFR1d2 by binding histidines His147 and His223 of each monomer. Three regions can be identified, Ile142-Thr149, Glu201-Leu204 and Thr222-Arg224, involved either in metal recognition and or in dimer formation. (TIF) [file pone.0167755.s001.tif]

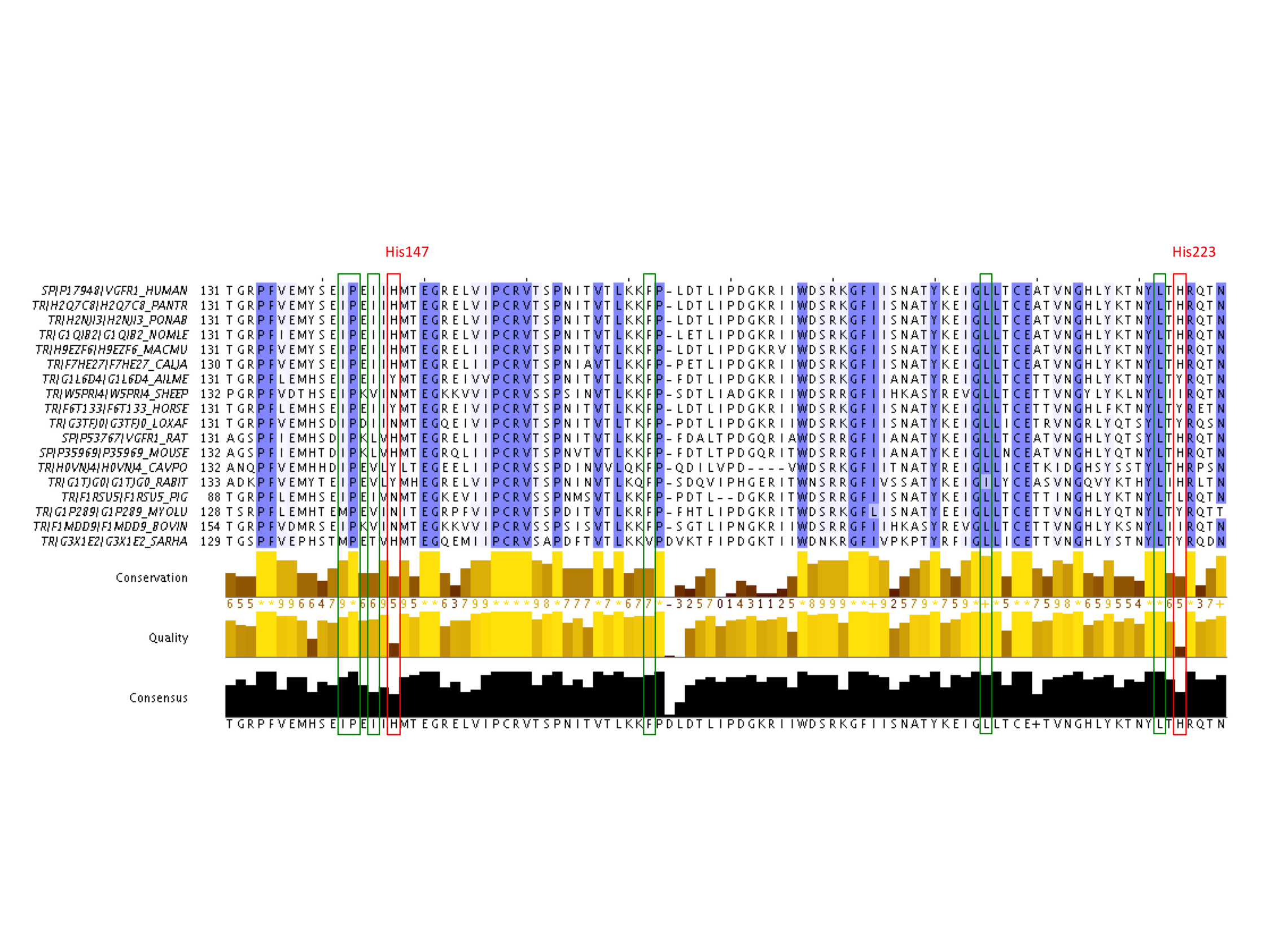

Supplement: S2 Fig — The residues encompassing the dimerization site are in green boxes and the His147 and His223 homologous are in red boxes. (TIF) [file pone.0167755.s002.tif]

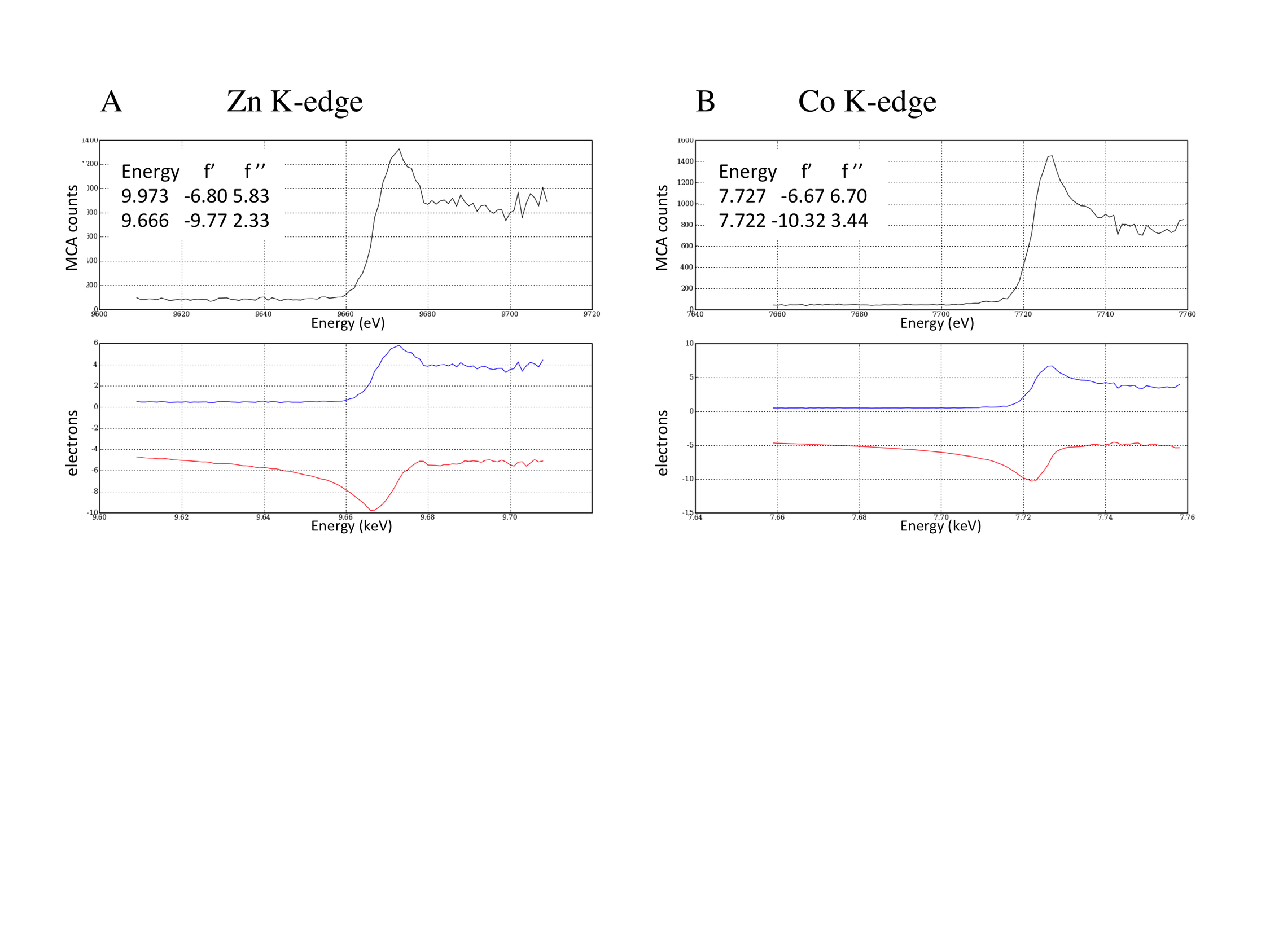

Supplement: S3 Fig — (A) hVEGFR1d2 crystallized in C2221 space group in the presence of 4 mM Laxaphycin B. (B) hVEGFR1d2 crystallized in P1 space group in the presence of 10 mM CoCl2. The spectra revealed a unique anomalous signal for each crystal form that corresponds to zinc K-edge (A) or to cobalt K-edge (B). (TIF) [file pone.0167755.s003.tif]
